# Response to Salaspuro and Lachenmeier, 2020, letter to the editor in Archives of Toxicology

**DOI:** 10.1007/s00204-020-02898-w

**Published:** 2020-09-07

**Authors:** Andrea Hartwig, Gerhard Eisenbrand

**Affiliations:** 1 Department of Food Chemistry and Toxicology, Institute of Applied Biosciences (IAB), Karlsruhe Institute of Technology (KIT), Adenauerring 20a, 76131 Karlsruhe, Germany; 2 Retired Senior Professor for Food Chemistry and Toxicology, Kühler Grund 48/1, 69126 Heidelberg, Germany

In their LETTER TO THE EDITOR “Unique human cancer model for acetaldehyde based on Mendelian randomization”, Mikko Salaspuro and Dirk W. Lachenmeier (Salaspuro and Lachenmeier 2020) comment on our recent review “Mode of action-based risk assessment of genotoxic carcinogens” (Hartwig et al. 2020). We are pleased that the authors support our conclusion that in case of ethanol and acetaldehyde, local concentrations in the digestive tract seem to play a major role in the development of cancer (Hartwig et al. 2020). They highlight the crucial role of local acetaldehyde concentrations in ethanol-related upper digestive tract cancers. Salivary acetaldehyde concentrations after ingestion of ethanol with food and/or alcoholic beverages primarily are governed by microbial metabolism of the microbiome in the oral cavity. According to this model, absorbed ethanol after systemic distribution diffuses back to saliva from blood to become converted to acetaldehyde. Kinetics are characterized by an instant increase of salivary acetaldehyde, followed by a long-term phase, lasting as long as ethanol is present in the circulation and excreted into saliva. These findings are in line with epidemiological data, showing dose-dependent higher risks (ORs) for oropharyngeal cancer in ALDH2 deficients compared to ALDH2-proficient humans. We largely agree with this rational. However, as outlined in our review in more detail, ethanol as well as acetaldehyde are generated also endogenously, e.g., within amino acid metabolism and thus may contribute to salivary exposure, an aspect to be also considered in quantitative risk assessment. Moreover, in addition to its genotoxicity, the potential of acetaldehyde to cause tissue irritation may contribute a substantial promotional effect.

The relevance of the oral cavity microbiome in generating potentially harmful metabolites has already been recognized more than 50 years ago, with the discovery of microbial nitrite formation from salivary nitrate. Salivary nitrate directly reflects plasma nitrate that is greatly governed by dietary uptake, as summarized in DFG (2014). However, despite many years of research, the potential health risk resulting from the endogenous formation of carcinogenic *N*-Nitroso compounds still is not clear up to the present time. It appears timely, therefore, to resume research, on the potential influence, and mutual interaction, of such compounds in saliva on human cancer risk.
